# Business Venturing in Regulated Markets—Taxonomy and Archetypes of Digital Health Business Models in the European Union: Mixed Methods Descriptive and Exploratory Study

**DOI:** 10.2196/65725

**Published:** 2025-01-09

**Authors:** Sascha Noel Weimar, Rahel Sophie Martjan, Orestis Terzidis

**Affiliations:** 1 Institute for Entrepreneurship, Technology Management and Innovation (EnTechnon) Karlsruhe Institute of Technology (KIT) Karlsruhe Germany

**Keywords:** digital health, telemedicine, mobile health, business model, European Union, classification, archetypes, medical device regulations, mobile phone, artificial intelligence, AI

## Abstract

**Background:**

Digital health technology (DHT) has the potential to revolutionize the health care industry by reducing costs and improving the quality of care in a sector that faces significant challenges. However, the health care industry is complex, involving numerous stakeholders, and subject to extensive regulation. Within the European Union, medical device regulations impose stringent requirements on various ventures. Concurrently, new reimbursement pathways are also being developed for DHTs. In this dynamic context, establishing a sustainable and innovative business model around DHTs is fundamental for their successful commercialization. However, there is a notable lack of structured understanding regarding the overarching business models within the digital health sector.

**Objective:**

This study aims to address this gap and identify key elements and configurations of business models for DHTs in the European Union, thereby establishing a structured understanding of the archetypal business models in use.

**Methods:**

The study was conducted in 2 phases. First, a business model taxonomy for DHTs was developed based on a systematic literature review, the analysis of 169 European real-world business models, and qualitative evaluation through 13 expert interviews. Subsequently, a 2-step clustering analysis was conducted on the 169 DHT business models to identify distinct business model archetypes.

**Results:**

The developed taxonomy of DHT business models revealed 11 central dimensions organized into 4 meta-dimensions. Each dimension comprises 2 to 9 characteristics capturing relevant aspects of DHT business models. In addition, 6 archetypes of DHT business models were identified: administration and communication supporter (A1), insurer-to-consumer digital therapeutics and care (A2), diagnostic and treatment enabler (A3), professional monitoring platforms (A4), clinical research and solution accelerators (A5), and direct-to-consumer wellness and lifestyle (A6).

**Conclusions:**

The findings highlight the critical elements constituting business models in the DHT domain, emphasizing the substantial impact of medical device regulations and revenue models, which often involve reimbursement from stakeholders such as health insurers. Three drivers contributing to DHT business model innovation were identified: direct targeting of patients and private individuals, use of artificial intelligence as an enabler, and development of DHT-specific reimbursement pathways. The study also uncovered surprising business model patterns, including shifts between regulated medical devices and unregulated research applications, as well as wellness and lifestyle solutions. This research enriches the understanding of business models in digital health, offering valuable insights for researchers and digital health entrepreneurs.

## Introduction

### Background

In recent years, digital health technologies (DHTs) have seen significant advancements, with some apps becoming prescribable by physicians and reimbursable by health insurers, thus making them accessible to patients at no additional cost via their smartphones [1]. Germany has pioneered this approach, and several other countries have since adopted similar measures [2]. This development marks a significant step forward in DHT adoption. However, the potential value of DHTs extends beyond mobile apps. They can support physicians in diagnostic and treatment decisions, streamline administrative tasks, or support clinical trials, among other functions [3,4]. With health care systems worldwide struggling with sustainability issues [3] due to aging populations and increasing numbers of chronic diseases [5], DHTs play a crucial role in delivering value, enhancing the quality of care, and reducing rising health care costs [6].

DHTs encompass systems that integrate computing platforms, connectivity, software, and sensors for health care and related use [7]. These digital technologies primarily consist of stand-alone software solutions. Despite their promise, integrating DHTs into health care systems presents significant challenges due to the complexity and stringent regulations of these systems. The European Union (EU) recently introduced new, more stringent medical device regulations [8]. Medical devices for human use in the EU are now governed by Regulation (EU) 2017/745 on medical devices (MDR) or Regulation (EU) 2017/746 on in vitro diagnostic medical devices (IVDR) [8,9]. These regulations aim to ensure high standards of quality and safety for medical devices [8]. Consequently, some DHTs are classified as medical devices, while others do not meet this criterion [7]. In addition, various countries in the EU are establishing new reimbursement pathways specific to DHTs [2], such as the fast-track approach for digital health apps in Germany mentioned previously [1]. This development opens new revenue options for DHT businesses. The complexity of health care systems is further compounded by the broad stakeholder landscape that includes health insurers, health care providers, and patients, among others [10]. New ventures in the emerging DHT segment face substantial challenges in bringing their solution to market [11]. These challenges include meeting the stringent requirements of the medical device regulations, such as the need to build a quality management system and collect clinical evidence [9]. These activities demand significant additional time and costs [8]. Furthermore, the revenue model poses a challenge because consumer willingness to pay is often low, with many accustomed to free health care services [12]. Reimbursement pathways for digital health are often unclear and vary between several EU countries [2]. Moreover, high-quality, real-world evidence is often required to secure reimbursement [1].

To overcome these challenges and realize the full potential of DHTs, establishing a well-defined and sustainable business model is one of the crucial elements for success [13]. A DHT business model can help to cocreate and formulate a set of critical success factors [5]. Although start-ups have limited resources, they can leverage their structural flexibility to explore new market segments, thereby playing a pivotal role in introducing and adopting innovative business models [14]. This innovation is particularly important because, although medical technologies are constantly introduced to the market, they rely on less inventive business models [15]. Consequently, emerging business models built on DHTs can address the shortcomings of traditional medical technologies, overcoming barriers to commercialization. Therefore, a structured understanding can help identify sustainable and innovative DHT business model types. This understanding can also support the development process of business models at different stages [16]. However, there is a significant gap in the structured understanding of the business models within the DHT domain. In addition, there is a lack of research on how medical device regulations impact digital business models despite these regulations having a considerable influence [17].

### Objectives

This study aims to identify key elements and configurations of business models for DHTs and, by doing so, provide a structured understanding of the archetypal business models in use. Given the variations in medical device regulations across countries, this study focuses on the EU with a common medical device regulatory framework. To achieve this goal, we formulated 2 research questions (RQs):

- RQ1: What are the key observable elements of DHT business models in the EU?
- RQ2: Which archetypes of DHT business models in the EU can be identified?

To answer these RQs, we developed a DHT business model taxonomy based on the digital health business model literature, data from real-world start-ups, and expert interviews. Subsequently, we derived DHT business model archetypes through cluster analysis. This approach provides a systematic classification of DHT business models and a simplified overview that establishes a common language. In addition, this study contributes to business model theory in complex domains by describing key components, the influence of the regulations, and drivers for business model innovation. In practical terms, this clear structure, real-world cases, and archetypal descriptions can assist digital health entrepreneurs in identifying suitable business models for their DHTs. It may also aid venture capitalists and investors in systematically analyzing digital health ventures.

The remainder of this work is structured as follows: the next subsection provides an overview of current research on business models in digital health. The Methods section outlines the research methodology followed, which is based on 2 main research phases. The Results section presents the developed business model taxonomy and archetypes. Finally, the Discussion section discusses the results and concludes the study.

### Related Work

Digital health has emerged as an umbrella term to describe the practice of using information and communication technology to address health care needs [18]. The main components of digital health include eHealth, which encompasses internet and web technologies used in health care delivery; mobile health (mHealth), which focuses on mobile devices to administer health care services; and telemedicine, which involves remotely connecting physicians and patients [19,20]. The digital technologies used in the context of digital health are referred to as DHTs. DHTs, which are regulated as medical devices, are known as software as a medical device [7]. The term *software as a medical device* refers to software intended to be used for at least 1 medical purpose that is performed without being part of a hardware medical device [21]. The intended medical purpose, declared by the medical device manufacturer, qualifies a DHT as a medical device [9]. The EU definitions of a medical device, in vitro diagnostic medical device, medical purpose, and other relevant definitions in this context are provided in Table S1 in Multimedia Appendix 1.

A business model can be described as the mechanism by which a business creates, delivers, and captures customer value [22]. Osterwalder [23] defines the business model as a strategic plan translated into a conceptual blueprint, positioning the strategy as one layer removed from the business model itself. In this context, the business model lies between the business strategy and the business processes and can be used as a tool of alignment through explicit depiction of the business logic [13]. It implements strategies and reflects the realized strategy [24]. The broad scope of business model research includes several subareas, such as research on business model components, development tools, and taxonomies [16]. Among development tools, the business model canvas is widely used to support the development process [25].

Existing literature discusses business models of digital health and related subcategories, such as telemedicine, eHealth, and mHealth. Some conceptual work exists in this domain [14,26-28]; for instance, Pascarelli et al [28] and Velayati et al [27] conducted systematic literature reviews to identify key components of digital health and telemedicine business models. Much of the research is empirical, describing elements of business models in digital health, telemedicine, and eHealth [29,30]. Some studies use the business model canvas to frame their empirical research [31-34], while others present their own frameworks [35,36]. Sterling and LeRouge [30] identified value proposition, key processes, key resources, and the profit formula as key components, while Gehde et al [4] focused on the technologies used to distinguish between business models.

Classification is the broad concept of organizing knowledge [37]. Under this scope, taxonomies create a structure and organization of a knowledge field and can help to navigate it [38]. While taxonomies are typically built inductively from empirical data, the term *typology* often denotes deductively derived classifications [16], although these concepts are sometimes used interchangeably [38].

Some taxonomies exist in the realm of digital health [39-42]. These taxonomies primarily emphasize technical aspects and lean toward telemedicine, but they also provide some useful criteria in the context of business models, such as technology [40], care sectors [41], or data types [42].

Business model taxonomies categorize business models based on their unique features [16] and aim to formulate ideal types of businesses [43]. These types of taxonomies have recently seen increased attention [16]. A few studies have focused on digital health business model taxonomies; for instance, a validated taxonomic business model description for telemedicine highlights the dimensions of value proposition, value cocreation, value communication and transfer, and value capture [44]. Three patterns of business models were identified. Other specific business model taxonomies include those for specialized mHealth segments such as maternal and baby care [45] or Middle Eastern telemedicine [46].

In conclusion, the focus and results of the studies vary depending on the digital health domain and research methods used. While some research has explored the components of digital health business models, a comprehensive and systematic classification that accounts for unique features, such as medical device regulations, and is based on extensive empirical data remains lacking.

## Methods

### Overview

**Figure 1:**
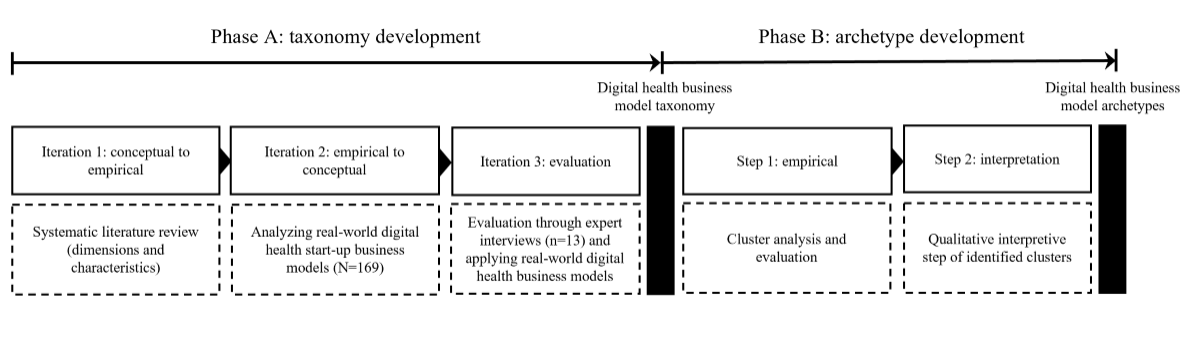
Overview of the research design, which was organized into 2 main phases: taxonomy development and archetype development. Each phase encompassed multiple iterations and steps.

Our research design comprises 2 phases, as illustrated in Figure 1. These phases include the development of the taxonomy followed by the archetype development. This research design is inspired by similar work on business model taxonomy development [47-49].

### Phase A: Taxonomy Development

#### Overview

In the initial phase, we followed the iterative taxonomy development process proposed by Nickerson et al [38], incorporating recent enhancements by Kundisch et al [50]. The systematic process developed by Nickerson et al [38] is well established for its ability to integrate both empirical (inductive) and conceptual (deductive) approaches, allowing for iterative refinement until specified ending conditions are met [38]. Therefore, taxonomy design can be systematized, producing comprehensive and robust results. Kundisch et al [50] extended the taxonomy development process proposed by Nickerson et al [38] with more emphasis on taxonomy evaluation. Therefore, we focus on the approach of Nickerson et al [38] and integrate the suggestions for evaluation proposed by Kundisch et al [50], similar to previous taxonomy development [49].

Following the recommendations of Nickerson et al [38], our first step was to define a meta-characteristic to guide the taxonomy design. We selected “key observable and unique elements of DHT business models in the EU” as our meta-characteristic. Furthermore, our ending conditions were based on the work of Nickerson et al [38] and can be found in Table S2 in Multimedia Appendix 1. We selected the V4 business model dimensions developed by Al-Debei and Avison [13] as a theoretical lens for the taxonomy design, which is a common approach in business model taxonomy development [43]. Consequently, we categorized all identified taxonomy dimensions into 4 meta-dimensions: value proposition (offering value structure), value architecture (technological and organizational architecture), value finance (financial setups and return), and value network (business and customer actors web) as defined by Al-Debei and Avison [13]. Building on these foundations, we set out to develop the taxonomy in 3 iterations. The first iteration followed a deductive, conceptual-to-empirical approach grounded in a literature review. By contrast, the second iteration adopted an inductive, empirical-to-conceptual approach and relied on real-world observations, while the third iteration focused on evaluating the taxonomy through expert interviews. Throughout these iterations, we were mindful of potential biases related to our methods. Systematic literature reviews can be prone to publication selection biases, while qualitative research may introduce interpretive biases. To mitigate the risks of individual methods, we used a mixed methods approach and discussed results within the team to enhance objectivity. This process enabled us to incorporate diverse perspectives, resulting in a more comprehensive and robust taxonomy [38].

#### Iteration 1: Systematic Literature Review

**Figure 2:**
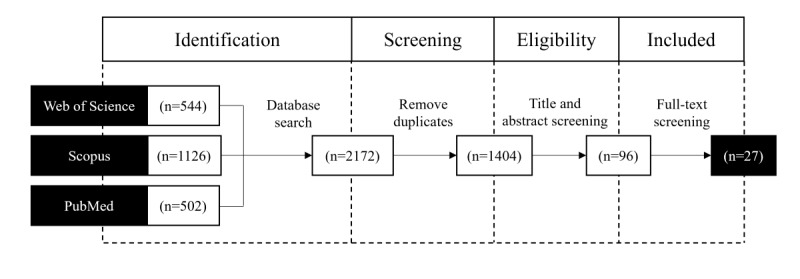
Visualization of the publication selection process as part of the systematic literature review, which involved searching 3 databases, applying a targeted search string, and using inclusion criteria to identify relevant studies.

The first iteration of the taxonomy development was based on a systematic literature review [51,52] to identify initial taxonomy dimensions and characteristics. A systematic literature review is a transparent and reproducible methodology for identifying relevant scientific work in a field [51]. This process involves planning the review, identifying and evaluating studies, extracting and synthesizing data, and disseminating the review findings [51]. An overview of the publication selection process is illustrated in Figure 2. To identify relevant publications, we conducted a comprehensive search in February 2024 across 3 major databases: Web of Science, Scopus, and PubMed. We applied an extensive search string to titles, abstracts, and keywords: (“digital health*” OR “mobile health*” OR “mhealth*” OR “m-health*” OR “ehealth*” OR “e-health*” OR “telemedicine*” OR “tele-medicine*” OR “telehealth*” OR “tele-health*” OR “software as a medical device*” OR “digital therapeutic*”) AND (“business model*” OR “taxonom*” OR “archetyp*”). We did not limit the search period. This approach yielded a total of 2172 potential publications. After removing duplicates, titles and abstracts were scanned, and 3 inclusion criteria were applied: publications must (1) mention potential business model dimensions of digital health, (2) be written in English, and (3) be peer reviewed. Applying these inclusion criteria to the 2172 potential publications resulted in 96 (4.42%) publications. A subsequent full-text screening of these 96 publications narrowed this down to 27 (28%) final publications [4,6,19,26,28,30,32-36,39-42,44-46,53-61]. Almost half of the papers (13/27, 48%) use qualitative or conceptual research methods. A comprehensive list of the included publications, along with their study objectives, research methods, and key dimensions, is presented in Table S3 in Multimedia Appendix 1.

The knowledge gained from these publications was incorporated as a theoretical foundation into the subsequent analysis. Common themes were identified and then conceptualized as dimensions and characteristics based on the V4 meta-dimensions [13].

The meta-dimension *value proposition* was frequently highlighted as a central element of the business models in digital health [28,44], with the most commonly mentioned dimensions being the *main purpose* [4,44,45] and the *target user*, referring to the primary customer of a digital health company [6,33].

Under the meta-dimension *value architecture*, digital technology [44] and regulations [36,45] were identified as key business model elements. In addition, data often drive digital business models [26,33], leading us to introduce the dimensions *enabling technology*, *regulations*, and *data type*. These dimensions incorporate key resources [30] and dominate key activities [28,34] relevant to the business model. Recognizing the importance of embedding a business model into the health care system, we created the dimension *medical specialty level* [35,41] as the final dimension of *value architecture*.

For the third meta-dimension, *value finance*, we incorporated *paying entity* [44,45] to distinguish between users and payers, often different health care entities. Health care–specific reimbursement options were mentioned in the literature, where health insurers cover costs for digital health solutions [28,35]. This led to the establishment of the dimension *reimbursement*. In addition, *funding* was established as a further dimension [6,28].

In the final meta-dimension, *value network*, partnerships along the value chain were identified as part of the business model [26,28], prompting us to introduce the dimension *value chain partnerships*. Furthermore, digital health also plays a crucial role in connecting users through digital platforms [35,44,45], leading to the establishment of the dimension *platform interaction type*.

These initial dimensions were frequently cited in the literature; however, some variability between studies was observed, likely due to differences in research methods and the specific focus areas of the studies.

#### Iteration 2: Real-World Digital Health Business Models

**Figure 3:**
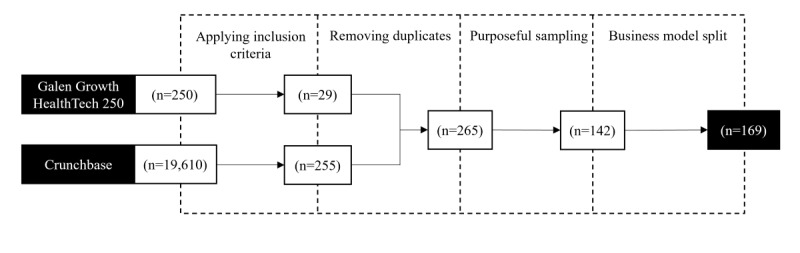
A summary of the selection process for real-world digital health technology business models, incorporating 2 primary sources for ventures and applying specific inclusion criteria.

Transitioning from the conceptual-to-empirical step, we adopted an empirical-to-conceptual approach for the second iteration of taxonomy development by analyzing real-world DHT business models. A fruitful approach is to analyze start-up business models because they often have novel digital business models and good availability and narrowness of data [43]. We used the HealthTech 250 list by Galen Growth [62] and the business intelligence platform Crunchbase to identify suitable start-ups to build our dataset. The HealthTech 250 list comprises 250 early-stage digital health ventures. On Crunchbase, by setting the industry group to “health care” and limiting the Crunchbase Rank, which is a measure of a company’s prominence [63], to 100,000 companies, we received an initial 19,610 results. To refine these results and focus on start-ups, inclusion criteria were established. First, the start-ups should be located in the EU and target this market. As medical device regulatory frameworks vary between countries, the EU enables the study of a common regulatory framework. Second, the solution developed by the venture should fit into the scope of DHTs. Third, the company should not be older than 10 years [64]. Next, the maximum number of employees should not exceed 250, aligning with the EU’s commonly defined upper limit for small- and medium-sized enterprises [65]. Finally, the company must be privately held and should not have merged with, or been acquired by, another entity. These criteria, which should ensure that the companies have a clear, identifiable business model and can be considered start-ups, were applied to both the HealthTech 250 list and the Crunchbase search. Afterward, we removed duplicates and performed purposeful sampling to create a balanced dataset and avoid overrepresenting specific business model types [66]. The complete list of companies, with relevant metadata, is shown in Table S4 in Multimedia Appendix 1. Next, we randomly scanned companies from our sample by reviewing their websites and other publicly available information to verify existing dimensions and characteristics and identify additional ones based on our meta-characteristic. If more than 1 applicable business model was identified for a company, each business model was considered separately, as depicted in the last step in Figure 3.

This process led to the identification of 12 dimensions and 64 characteristics. Finally, theoretical saturation was achieved after no new business models were identified for >20 companies in a row, and the second taxonomy development iteration was concluded.

#### Iteration 3: Qualitative Evaluation

It has been emphasized that the evaluation of a taxonomy is a crucial element of the taxonomy design that should not be ignored [50,67]. On the basis of this understanding, we extended the taxonomy development process proposed by Nickerson et al [38] with a third iteration, with the goal of triangulating the previous quantitative research approach with a qualitative one. Hence, we evaluated the previously developed taxonomy qualitatively through semistructured expert interviews. Interviews allow the collection of detailed and context-rich data, offering deep insights into a research topic [68]. We built our qualitative evaluation on commonly mentioned and applied criteria for taxonomy evaluation: completeness, simplicity, understandability, and robustness [50]. On the basis of these criteria and following the questions proposed by Szopinski et al [67] for taxonomy evaluations, we prepared an interview guideline. Thirteen experts from both academia and industry, with relevant expertise in digital health, business models, or taxonomy development were selected to participate. Each interview was conducted using videoconference software and was recorded and transcribed. An overview of the experts’ roles, expertise, and interview durations (total duration: 407 min) is presented in Table 1.

**Table 1:** Overview of the interviewed experts’ roles, expertise, and interview durations.

ID	Role	Expertise	Duration (min)
E1	Research group leader	Digital health and taxonomies	25
E2	Senior consultant	Taxonomies and business models	30
E3	Start-up consultant	Digital health and business models	47
E4	Cofounder	Digital health and business models	37
E5	Cofounder	Digital health and business models	28
E6	Cofounder	Digital health and business models	35
E7	PhD student	Digital health and business models	25
E8	Chief strategy officer	Digital health and business models	26
E9	Pharmaceutical industry consultant	Digital health and business models	38
E10	Venture capitalist	Digital health and business models	37
E11	PhD student	Digital health and business models	20
E12	Junior professor	Taxonomies and business models	25
E13	PhD student	Taxonomies and business models	34

The transcripts were inductively analyzed using the Gioia methodology [69]. On the basis of this analysis, we used the taxonomy operators *adding*, *renaming*, *merging*, and *removing* to rework dimensions or characteristics [50]. A table with the detailed changes after conducting the expert interviews can be found in Table S5 in Multimedia Appendix 1. We applied the updated taxonomy to our previously derived sample of DHT start-ups, resulting in no further changes to the taxonomy. To validate our findings, 2 additional raters coded a subsample (10%) of the real-world cases. The resulting Fleiss κ value of 0.79 indicates substantial agreement with the original classification [70]. This concluded the taxonomy development, with all ending conditions met (refer to the Results section for the final taxonomy version).

### Phase B: Archetype Development

In the second phase, a cluster analysis was performed to develop archetypes of DHT business models. This method groups observations based on their similarities [71] and is the dominant mechanism for generating business model archetypes [43]. It is an objective and data-driven approach, thereby reducing potential biases. In addition, several clustering methods are available that enable fine-tuning and ensure that the clusters reflect meaningful and interpretable business model archetypes. Various approaches and methods for cluster analysis exist, raising questions regarding which similarity or dissimilarity measure to choose and which algorithm to use [72]; for instance, iterative partitioning methods such as k-means are considered superior to hierarchical algorithms [72], but they require predefined cluster numbers, which hierarchical algorithms do not require [72]. To overcome this limitation, we followed the 2-stage clustering approach proposed by Punj and Stewart [72].

In the first stage, we used the method proposed by Ward [73] to perform agglomerative hierarchical clustering. We used the simple matching coefficient as a similarity measure suitable for binary datasets [48]. The resulting clusters were visualized in a dendrogram (Figure S1 in Multimedia Appendix 1). Clusters that merge at greater heights in the dendrogram are more dissimilar. Therefore, cluster numbers were deemed reasonable in the range of 2 to 6 because the height differences between merge points dropped significantly beyond 6 clusters.

In the second stage of the cluster analysis, we performed k-means clustering with various cluster numbers to identify the optimal number [47]. The elbow rule indicated that 6 clusters were an optimal solution (Figure S2 in Multimedia Appendix 1). In addition to k-means, we conducted a similar analysis using algorithms such as k-modes, which are suitable for binary datasets. On the basis of these analyses, we examined the number of cases per cluster and discussed the interpretability and explanatory power of the clusters within the team. The 6-cluster solution of the k-means algorithm was identified as the strongest, with each cluster being well separated and interpretable (Table S6 in Multimedia Appendix 1). The average silhouette score of the solution was 0.35, suggesting a rather weak but substantial cluster structure in the data [74]. Social science data frequently lack strong natural groupings [75], and similar results have been observed in previous business model taxonomy research [48]. Therefore, we interpret the result as a good clustering outcome for the diverse and complex configurations of DHT business models observed in our study.

Finally, we conducted a qualitative interpretive step to interpret the results and the corresponding clusters by inspecting the frequency distributions of each characteristic within the clusters [75]. The frequency distributions for each cluster are presented in Table S7 in Multimedia Appendix 1.

### Ethical Considerations

The qualitative data in this study, collected through interviews, have been deidentified to protect participant privacy and confidentiality. All interviewees provided informed consent for the use of deidentified data in this scientific publication. The research was conducted in accordance with the relevant ethics guidelines, and it was exempt from human participant ethics review.

## Results

### A Taxonomy of European DHT Business Models

The resulting taxonomy is presented as a morphological box in Figure S3 in Multimedia Appendix 1. In total, 11 dimensions were identified. Some dimensions are exclusive, allowing only 1 characteristic to be assigned, while others are nonexclusive, allowing multiple characteristics to be assigned. All characteristics in Figure S3 in Multimedia Appendix 1 are sorted alphabetically. The key elements of the taxonomy, based on 4 meta-dimensions, are discussed herein. A detailed description of each dimension and characteristic is presented in Table S8 in Multimedia Appendix 1, and an example of how a DHT business model can be categorized within the taxonomy is presented in Table S9 in Multimedia Appendix 1.

The *value proposition* meta-dimension outlines how a company offers value [13]. In DHT business models, 9 *main purposes* have been identified, indicating the type of value delivered. These purposes are divided into 2 main groups: those with a medical purpose and those without (refer to Table S1 in Multimedia Appendix 1 for the definition of *medical purpose*). Characteristics with a medical purpose include *diagnosis or prediction*, *monitoring support*, and *treatment or interventional purpose*. All these characteristics are related to specific medical conditions. The *treatment or interventional purpose* characteristic also encompasses preventive and rehabilitation interventions [76,77]. Characteristics without a medical purpose include *administrative support* and *information delivery or education*, among others. DHT business models can have multiple *main purposes*, including combinations of medical and nonmedical purposes. It is crucial to identify the primary source of value when selecting the *main purpose*. Another dimension of the *value proposition* meta-dimension is the *target user*, encompassing *patients or private individuals* and stakeholders directly involved in health care delivery, such as *health care providers*. Another target user is *indirect health care commercial firms*, such as pharmaceutical and medical technology companies or even *non–health care commercial firms*. Finally, some companies collect data to provide clinical and nonclinical *evidence*. Here, some companies base their evidence on established medical literature, while others provide real-world evidence, for example, through randomized controlled trials.

The *value architecture* meta-dimension describes the technical and organizational infrastructure of the business model [13]. This meta-dimension encompasses the integration of the business model into health care systems, considering the *level of care*, such as *primary*, *secondary*, or *tertiary care* [78,79]. This describes whether the value is generated through integrating solutions at the initial points of contact with health care systems or, alternatively, within highly specialized care settings. In addition, this meta-dimension includes the EU *medical device regulatory framework*, which comprises the *medical device regulation* covering most regulated DHTs with a medical purpose and the *in vitro diagnostic medical device regulation* for DHTs with a medical purpose using primarily biological data derived from human biological materials [9]. In addition, it recognizes the existence of *nonmedical devices* without a medical purpose. Digital business models are fundamentally driven by data as a key resource and often are built on an *enabling technology*. In total, 9 *data types* were identified, ranging from *medical imaging data* derived through x-ray imaging or magnetic resonance imaging to *physiological parameter data* captured using sensors to measure bodily functions. Some of the data are reported directly by patients or involve tracking user behavior, summarized under the dimension of *user activity or patient-reported data*. Examples of enabling technologies include *artificial intelligence*, *cloud or network technology*, and *sensor technology*.

*Value finance* is the part of the business model that focuses on financial setups and returns [13]. Therefore, the taxonomy includes the *paying entity*, which can be the *patient or other private individuals* such as caregivers, *health care providers*, or even companies indirectly involved in health care delivery. *Reimbursement* plays a vital role in the revenue model, with *health insurers and governments* covering expenses for DHTs, depending on the country-specific *reimbursement* pathways [2].

Finally, the *value network* is defined as the web of business and customer actors [13]. A key aspect is the *user interface*, which shapes how users interact with the solution. In addition, the business model taxonomy covers stakeholder interactions. Digital platforms can engage both internal and external actors [80], as illustrated in the dimension of *stakeholder interaction type*. This category examines network effects, focusing on user and company interactions facilitated by the solution. It assesses whether users engage with each other through the solution and how the companies themselves interact with their customers using the solution.

### Archetypes of DHT Business Models

#### Overview

Six archetypes of DHT business models were identified. The number of business models in these clusters ranges from 18 (10.7%) to 36 (21.3%) out of 169 business models. The frequency distribution for all characteristics of the archetypes is shown in Table S7 in Multimedia Appendix 1. Through an interpretive step, we analyzed the archetypes and assigned the following labels: administration and communication supporter (A1), insurer-to-consumer digital therapeutics and care (A2), diagnostic and treatment enabler (A3), professional monitoring platforms (A4), clinical research and solution accelerators (A5), and direct-to-consumer wellness and lifestyle (A6). Table 2 provides an overview of the archetypes, and the subsections that follow offer a comprehensive summary of the main findings for each cluster.

**Table 2:** Concise descriptions of the 6 business model archetypes identified through cluster analysis, along with corresponding examples.

ID	Archetypes	Distinguishing characteristics	Examples of underlying DHTs^a^
A1	Administration and communication supporter	Business models around tools assisting with administrative tasks (eg, scheduling and report writing) and patient interactions Not medical devices; often using cloud, network technology, and artificial intelligence; efficiency evidence not typically provided Health care providers pay for these nonreimbursable solutions; interfaces are mainly mobile apps and web applications	Automated medical report writing, appointment management tools, and telehealth video consultation
A2	Insurer-to-consumer digital therapeutics and care	Business models around supporting digital treatment of medical conditions, used mainly by patients and private individuals Regulated as medical devices under the MDR^b^, providing mostly high clinical evidence (peer-reviewed studies) Focus on primary and secondary care, rely on health insurer reimbursement, and incorporate multimedia and patient-reported data; some act as support channels with the provider	Mobile app for treatment support of leaky gut, depression, or overweight
A3	Diagnostic and treatment enabler	Business models for solutions assisting physicians in diagnosis or treatment planning, sometimes including administrative support Regulated as medical devices under the MDR (medical imaging and physiological data) or the IVDR^c^ (data derived from human biological materials) Used and paid for by health care providers; use artificial intelligence; feature mobile apps or web applications; do not facilitate stakeholder interaction	Radiological imaging tools, treatment planning software, and in vitro diagnostic software to support the diagnosis
A4	Professional monitoring platforms	Business models based on digital platforms to monitor patients’ medical conditions, connecting health care providers with patients and private individuals Classified as medical products under the MDR, supported by clinical evidence Facilitate interuser interaction; integrate administrative support, telecommunication, and education; primarily paid for by health care providers, sometimes reimbursable	Physician platform to monitor progression of Parkinson disease based on mobile app and therapeutic exercise–monitoring platform
A5	Clinical research and solution accelerators	Business models around the acceleration of clinical research or the provision of raw technology for pharmaceutical companies, medical technology companies, and research institutions Some solutions are classified as medical devices under the MDR, while research applications are not regulated Paid for by indirect health care firms and research institutions, may or may not facilitate stakeholder interaction and include developer tools as well as mobile apps and web applications	Diagnostic tools based on artificial intelligence that medical technology companies can integrate and software to support clinical trial execution through data collection
A6	Direct-to-consumer wellness and lifestyle	Business models enabling information delivery and education for patients and individuals, with some telecommunication features and marketplaces Not classified as medical devices Include coaching through mobile apps and web applications; sometimes provided by medical clinics; targeting self-pay markets and corporate wellness; some are reimbursable and report nonclinical evidence	Mobile app for meditation, pregnancy support apps, and health and sports coaching mobile app

^a^DHT: digital health technology.

^b^MDR: Regulation (EU) 2017/745 on medical devices.

^c^IVDR: Regulation (EU) 2017/746 on in vitro diagnostic medical devices.

#### Administration and Communication Supporter (A1)

The first archetype comprises business models built on software tools designed primarily to assist health care providers with administrative tasks such as appointment scheduling, automated report writing, job matchmaking, professional education, and claims management. These administrative solutions are often combined with telecommunication modules that facilitate interactions between health care providers and patients or private individuals. Therefore, they also target private individuals. As the underlying solutions do not serve a medical purpose, they are not classified as medical devices, and companies typically do not provide evidence to support their efficacy. These business models operate across all levels of care. Communication is primarily enabled through cloud and network technology, with some solutions incorporating artificial intelligence. The revenue model predominantly involves health care providers who use and pay for these solutions, making them generally nonreimbursable for patients. The user interfaces are mainly mobile apps and web applications, and the solutions often connect health care providers with patients.

#### Insurer-to-Consumer Digital Therapeutics and Care (A2)

The second type of business model comprises software solutions that primarily support digital treatment and care through interventions for medical conditions. These business models target mainly patients or private individuals. Some of the business models additionally enable telecommunication with employees of the solution provider itself. They typically provide a high level of clinical evidence, such as through published peer-reviewed studies. The focus lies predominantly on primary and secondary care. Due to their medical purpose, solutions under this archetype are regulated as medical devices under the MDR. They incorporate multimedia data for educational purposes and capture user activity and patient-reported data. Most of the business models in this archetype rely on reimbursement through health insurers to generate revenue. For cases that are not reimbursed, patients or private individuals are payers.

#### Diagnostic and Treatment Enabler (A3)

Similar to the first archetype, the third business model archetype is mainly used and paid for by health care providers, specifically professionals such as physicians. In a few cases, these professional tools are reimbursed through health insurers or are paid for by patients and private individuals. They primarily assist physicians in the diagnosis or treatment of medical conditions. Although they sometimes include additional administrative support, they are regulated as medical devices under the MDR or the IVDR for their medical purpose. Business models under the MDR are substantially driven by medical imaging data, physiological parameter data, or medical laboratory results. By contrast, business models under the IVDR primarily use data derived from human biological materials, such as genomics and microscopic cell images. The business models are built on solid clinical evidence. This category also includes business models built on diagnosis software for patients and private individuals that share the same main purpose regarding diagnosis and prediction as professional tools and are nonreimbursable. The business models are often enabled through artificial intelligence, feature mobile app or web application interfaces, and do not facilitate stakeholder interaction.

#### Professional Monitoring Platforms (A4)

This archetype focuses on business models with the purpose of monitoring patients and their medical conditions through digital platform solutions that connect health care providers with patients or private individuals. Both the platforms for health care providers and the patient-side applications vary in complexity. Depending on the business model, several other purposes are integrated with the monitoring, such as diagnosis and prediction, administrative support, telecommunication, or information delivery and education. Monitoring is often achieved through the collection of user activity or, in many cases, through the collection of patient-reported data (mostly patient-reported outcome measures). The underlying solutions are classified as medical devices under the MDR and are supported by clinical evidence. Typically, the business model focuses on secondary or tertiary care applications. These platform business models facilitate interuser interaction by connecting patients and health care providers. Health care providers primarily bear the cost, but sometimes solutions are reimbursable.

#### Clinical Research and Solution Accelerators (A5)

The fifth archetype primarily targets indirect health care commercial firms, such as pharmaceutical or medical technology companies, as well as research institutions. The underlying solutions do not provide direct health care but serve as tools to accelerate drug discovery and clinical research by offering digital tools for study execution. This application occurs outside of direct medical care. In addition, some business models are built on offering raw technology that can be used by medical technology companies as part of their solutions. While some solutions are classified as medical devices under the MDR, others are not, because research applications do not fall under the MDR or the IVDR. The payers in this archetype are indirect health care commercial firms and research institutions. Some business models do not involve solution-side stakeholder interaction, while others facilitate interuser interaction, for example, through contact with study participants for clinical research. A distinctive feature of these business models is that the raw technologies are not only provided as mobile apps and web applications but also as developer tools.

#### Direct-to-Consumer Wellness and Lifestyle (A6)

The last archetype proposes value through information delivery and education for patients and private individuals. These can be simple, user-facing applications for general well-being, such as those providing guided meditation. Another type of value that is delivered by some of these business models is telecommunication, and a few offer marketplaces targeting patients and private individuals. A unique aspect of some of these wellness and lifestyle solutions is the interaction between the solution provider and users, where employees of the solution provider connect with users and coach them through mobile apps and web applications. Sometimes, the contact can be through a solution provider–operated medical clinic. Solutions related to this business model archetype do not mention specific medical purposes and are not classified as medical devices. While some solutions aim at the self-pay market, others target non–health care commercial firms that are interested in corporate wellness, making them reimbursable for patients and private individuals. In addition, some companies report nonclinical evidence from their solutions, which might help to convince health insurers to reimburse costs.

## Discussion

### Principal Findings

The objective of this study was to identify key elements of business models for DHTs, aiming to develop a structured understanding of the archetypal business models used in the industry. This goal was achieved through a 2-phase process. In the first phase, we developed a taxonomy of DHT business models [38,50]. The taxonomy was based on a systematic literature review and an analysis of 169 real-world business model cases. It was evaluated qualitatively through 13 expert interviews. In the second phase, we conducted a cluster analysis based on hierarchical and iterative partitioning clustering methods [72], which resulted in the identification of 6 archetypal clusters. These clusters were subsequently evaluated and interpreted. This resulted in interesting findings regarding the dominant business models used for DHTs and enabled the study of business model innovation in the DHT domain.

Primarily, we want to mention the cascading effect observed for the *medical device regulatory framework* dimension. Medical device regulations significantly impact the business model design if a company delivers a value proposition based on a medical purpose, which applies mainly to archetypes A2, A3, and A4. Through this, these regulations have a strong influence, requiring ventures to adapt their strategies based on available resources due to the additional time, costs, and potential partnerships needed. Many medical device companies in our sample of digital health start-ups (49/142, 34.5%) showcased proof of clinical evidence because the medical device regulations require demonstrating sufficient evidence. Some of the companies (7/142, 4.9%) based their evidence on the state of the art, while others (42/142, 29.6%) presented results from their own clinical studies. Furthermore, clinical partnerships and other quality and security certifications were listed on the company websites. Therefore, medical device regulations exert a strong influence on business models. While medical device regulations have the largest impact, other EU and local regulations also contribute to the cascading effect; for instance, the Artificial Intelligence Act recently adopted by the EU is relevant if the business model relies on artificial intelligence technologies that pose risks deemed sufficiently high. Thus, some companies must navigate a complex regulatory landscape to ensure compliance and sustainability when designing their business models.

Second, we identified 3 key drivers of business model innovation within the DHT domain. The first driver of business model innovation pertains to the dimensions of users and payers. Unlike traditional medical technology companies, which often develop business models centered around hardware-intensive technologies operated by health care providers, DHT business models can directly engage patients, private individuals, and even corporate employers. This is facilitated by the integration of mobile interfaces with cloud and network technologies, promoting innovative business models, particularly in archetypes A2, A4, and A6. The second driver is the advent of artificial intelligence and the rise of digital technologies, which enable unprecedented value delivery. These advancements facilitate the creation of business models that were previously unimaginable, significantly driving archetypes A4, A5, and A6. The third driver of business model innovation is the revenue model, customized to the target countries’ specific conditions. The self-payer market is more attractive in some regions, whereas others have established reimbursement pathways allowing cost coverage by 1 health insurer or multiple health insurers [2]. It has been observed that certain reimbursement pathways are exclusively available to medical devices or specific DHTs; for example, the reimbursement pathway for digital health applications in Germany [1] is limited to digital medical devices. This fosters new business models and drives innovation in archetypes A2 and A6. Companies in the study sample (26/142, 18.3%) engage in selective contracts with individual health insurers or use other pathways that facilitate the reimbursement of health care expenses. Therefore, the revenue model constitutes a critical element of DHT business models and must be tailored to the national context while ensuring alignment with other business model components. Start-ups should consider reimbursement pathways and possible related medical device requirements early in their strategy development.

**Figure 4:**
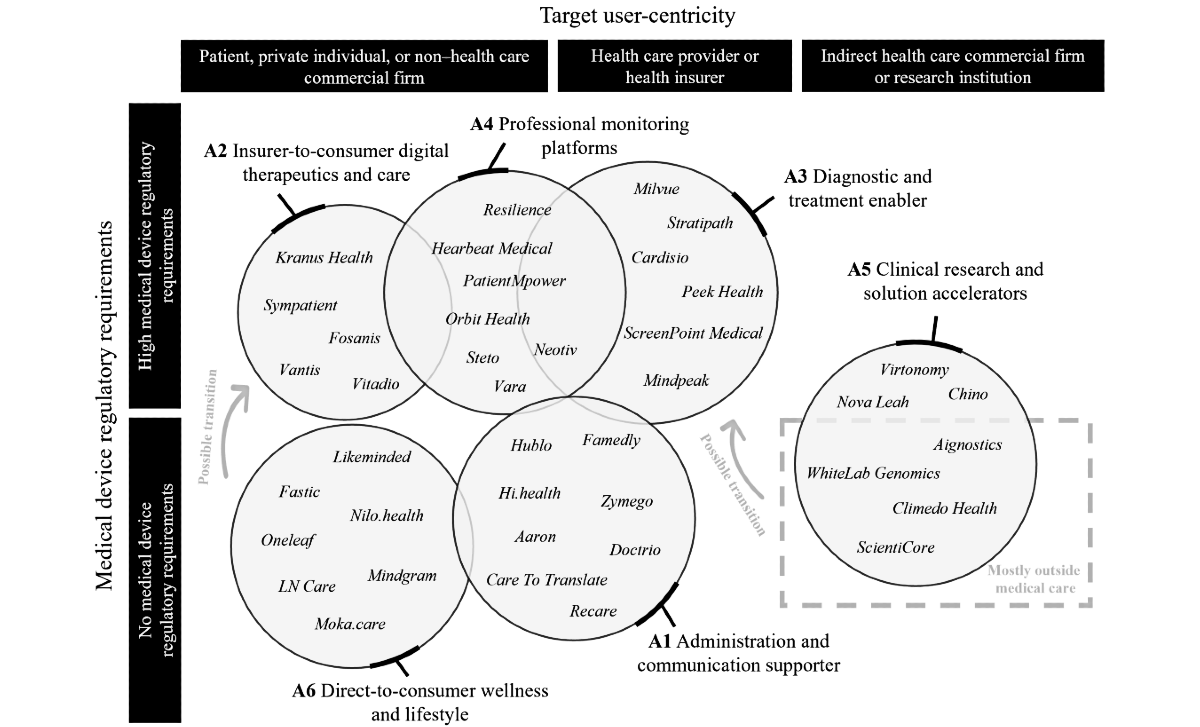
A visual representation of the 6 business model archetypes, each accompanied by example ventures organized by target user–centricity and relevant medical device regulatory requirements.

Finally, the relationships among the identified business model archetypes reveal interesting connections. To discuss these relationships, we categorize the 6 identified archetypes of business models based on their compliance with medical device regulations and their primary target users, as depicted in Figure 4. The solutions underlying the business model archetypes A2, A3, A4, and some in A5 are regulated as medical devices. Notably, A3, A4, and some A2 business models face stringent medical device regulatory requirements because they provide critical tools for health care providers and patients. A3 stands out because it is the only archetype that includes in vitro diagnostic medical devices regulated under the IVDR. It was noted that these business models often emerge from academia as transfer projects. While most health care providers typically use and cover the costs of these solutions, 1 particular business model stands out and deserves mention: in this model, patients pay for enhanced diagnoses through professional software, and health care providers receive a share of this payment as additional revenue. By contrast, the underlying solutions of A6 and A1 are not classified as medical devices. Notably, some companies within these archetypes, as well as A2 companies, operate at the intersection of regulated medical devices and unregulated wellness and lifestyle products [81]. This implies that these companies might initially adopt an A6 business model (no medical purpose and not subject to medical device regulations) and gradually transition to an A2 business model as they gather more real-world evidence (medical purpose and subject to medical device regulations). A5 business models play a unique role because they operate outside of direct medical care and are not subject to medical device regulations as research applications. Similar to the archetype A6, some companies adopting A5 business models can decide to progressively evolve into A3 business models over time as more real-world evidence is collected. In addition, a common approach is to combine A2 or A6 with A4. This combination involves a digital therapeutic, care application, or wellness and lifestyle solution that the patient uses, paired with a professional monitoring platform on the health care provider’s side, enabling monitoring through the patient-side applications. In such cases, patients, health care providers, or health insurers might be payers.

### Comparison With Prior Work

We developed a comprehensive taxonomy for business models based on 11 dimensions, following the V4 business model dimensions framework [13]. This taxonomy covers the broad space of DHT business models. Compared to prior studies in the field, which focused on specific subdomains of digital health, such as telemedicine [44] or maternal and baby segments in mHealth [45], our work confirms some of the previously identified dimensions, such as *target user*, *paying entity*, or *enabling technology*, as key resources. These dimensions are commonly used across various sectors to describe business models through taxonomies [44,45]. In addition to these dimensions, previous work has highlighted some taxonomy characteristics specific to the subdomains of digital health, such as medical devices under an mHealth category dimension [45], and overall purpose characteristics, such as diagnosis and therapy [44]. In our study, we describe the broad field of DHT business models. We have identified several new dimensions and characteristics that are highly relevant and specific to the field that had not been mentioned in previous business model taxonomy research. Notably, the *main purpose* dimension in our taxonomy enables the description of complex combinations of medical and nonmedical purposes related to the value proposition. In addition, the *medical device regulatory framework* has emerged as a critical dimension. This framework significantly impacts the entire business model, from value proposition to value architecture and value network. By focusing on the EU, we precisely delineated emerging business models shaped by medical device regulations and those that evolve around unregulated DHTs. Another dimension specific to DHT business models is the *evidence* dimension. In the context of DHTs, it is mandatory for medical devices to provide clinical evidence to enter the market. Interestingly, we found that even some nonregulated DHT business models provide evidence (in the form of nonclinical evidence) to facilitate reimbursement with health insurers and prove their value within the ecosystem. Developing reimbursement pathways specifically for digital health applications opens new revenue models. Consequently, the *reimbursement* dimension is also highly relevant for DHT business models. Finally, the health care *level of care* introduces an important consideration that has been previously overlooked: the necessity for business models to integrate into the existing health care system. An intriguing finding was that some business models focus on research applications within traditional research institutions and pharmaceutical companies. As these operate outside of medical care, their solutions do not need to comply with medical device regulations.

Our work is the first to delineate archetypes of DHT business models based on real-world observations in the EU while also considering medical device regulations. While some research has reported overarching areas of activities in digital health [4], typical value propositions in mHealth [29], or categorizations of patient needs that are addressed through different solutions [14], our work creates a clear image of archetypal business models used for stand-alone software in health care. The archetypes we identified align with the findings of Gehde et al [4]. The 13 areas of activity mentioned in their work are congruent with our findings and are covered by our archetypes. In addition, we mention here the work of the Digital Therapeutic Alliance [82], which, in partnership with Health Advances classified DHTs based on a technological perspective, identifying 8 major categories of DHTs. While the aforementioned work does not focus on the business models behind these solutions, our work offers suitable business models for the DHTs identified in that study. The DHT business models are inherently complex and exhibit some overlap. However, our archetypes effectively balance the need to simplify these complexities while still delivering valuable insights essential for designing business models in digital health.

### Limitations

Our research is not without limitations. First, the DHT business model landscape is rapidly evolving, with new start-ups emerging, new technologies being developed, and regulatory changes occurring. Our study provides a snapshot of the current business models used in the field. However, regulatory environments and business models are subject to change. Furthermore, our focus on observable business model elements of DHTs to derive our archetypes necessitated the exclusion of dimensions such as key partnerships, pricing strategy, and funding. However, these are fundamental to DHT business models as described in the literature and discussed in the expert interviews. In this context, we developed an empirically driven taxonomy to study business model archetypes. We argue that a taxonomy has limited capabilities to describe complex stakeholder and partnership configurations. The same applies to funding, which has a highly relevant temporal component. Finally, the taxonomy focuses on digital business models in the EU, which means we do not describe business models of hardware medical device manufacturers and have not incorporated other business model aspects that might be relevant in other continents.

### Contribution and Further Research

Theoretically, our work contributes to the systematic understanding of business models in the emerging DHT domain. By delineating common dimensions and characteristics of the field, we create a common language that paves the way for further research and helps scholars, such as those in business and management, health sciences, and information systems, to position their work within this landscape. The archetypes provide high-level descriptions of business models in this domain, offering a foundational framework for future studies. Furthermore, our dataset, which relies primarily on publicly available information from DHT companies, can serve as a basis for additional research.

On the practical side, the taxonomy we developed can assist entrepreneurs and start-ups in understanding key dimensions and characteristics relevant to DHT business models. An early informed understanding of the intricacies of a sustainable business model is fundamental for later success. In addition, the archetypes and strategic interpretation clarify the business models used in this domain, aiding entrepreneurs in identifying viable strategies based on common patterns. The data on the companies compiled in this work can serve as a starting point for competitor analysis or inspiration for developing their own business models. In addition to start-ups, established medical technology and pharmaceutical companies can benefit from a systematic understanding as they continually explore business model innovations to bring new solutions to the market. Investors, venture capitalists, and health insurers can also leverage this work to identify and validate viable business models. Finally, policy makers can use these insights when shaping new regulations.

Further research could delve deeper into the implications of funding and partnerships for the business model. In addition, the research could investigate business model configurations in other continents or even specific countries because reimbursement policies are specific to each country. Finally, further research could focus on business models of software and hardware combinations, such as software in a medical device.

### Conclusions

Digital health is a rapidly evolving field that is attracting increasing attention. In our work, we provide the first overview of business models for DHTs with a focus on the EU. We systematically describe business models in the field and elaborate on the influence of medical device regulations on the entire business model. Furthermore, we created a business model taxonomy based on a systematic literature review and real-world cases, which we evaluated qualitatively. In addition, we identified 6 archetypes of DHT business models through cluster analysis, describing key strategies and differences for each cluster. This allowed us to identify drivers for business model innovation in the DHT sector. These contributions offer a systematic understanding and common language to facilitate analyses and comprehension in this domain. Our findings could assist decision makers, especially start-ups, in positioning themselves and building sustainable business models in this evolving field.

## Appendix

Multimedia Appendix 1 Supplementary tables and figures.

## Abbreviations

DHT: digital health technology
EU: European Union
IVDR: Regulation (EU) 2017/746 on in vitro diagnostic medical devices
MDR: Regulation (EU) 2017/745 on medical devices
mHealth: mobile health
RQ: research question

## Data Availability

The datasets generated and analyzed during this study are available from the corresponding author on reasonable request.
