# Supplementary material for: Business Venturing in Regulated Markets—Taxonomy and Archetypes of Digital Health Business Models in the European Union: Mixed Methods Descriptive and Exploratory Study
Source: J Med Internet Res. 2025 Jan 9;27:e65725. doi: 10.2196/65725 (PMC11757981; doi:10.2196/65725)
Supplement: Multimedia Appendix 1 [file jmir_v27i1e65725_app1.pdf]

Multimedia Appendix. Supplementary tables and figures.

Table S1. Important definitions relevant in the context of digital health.

**Definition Digital Health Technology (DHT)**
System that uses computing platforms, connectivity, software, and sensors for healthcare and related uses. [1]
**Definition Software as a Medical Device (SaMD)**
Software intended to be used for one or more medical purposes that performs these purposes without being part of a hardware medical device. [1]
**Definition Software in a Medical Device (SiMD)**
Software that is used as an integral part of a specified hardware medical device or is intended to drive a hardware medical device. [1]
**European Union Definition Medical Device**
(1) ‘medical device’ means any instrument, apparatus, appliance, software, implant, reagent, material or other article intended by the manufacturer to be used, alone or in combination, for human beings for one or more of the following specific medical purposes:<br>— diagnosis, prevention, monitoring, prediction, prognosis, treatment or alleviation of disease,<br>— diagnosis, monitoring, treatment, alleviation of, or compensation for, an injury or disability,<br>— investigation, replacement or modification of the anatomy or of a physiological or pathological process or state,<br>— providing information by means of in vitro examination of specimens derived from the human body, including organ, blood and tissue donations,<br>and which does not achieve its principal intended action by pharmacological, immunological or metabolic means, in or on the human body, but which may be assisted in its function by such means.<br>The following products shall also be deemed to be medical devices:<br>— devices for the control or support of conception;<br>— products specifically intended for the cleaning, disinfection or sterilisation of devices as referred to in Article 1(4) and of those referred to in the first paragraph of this point. [2]

Continued from previous page
**European Union Definition In Vitro Diagnostic Medical Device**
(2) ‘in vitro diagnostic medical device’ means any medical device which is a reagent, reagent product, calibrator, control material, kit, instrument, apparatus, piece of equipment, software or system, whether used alone or in combination, intended by the manufacturer to be used in vitro for the examination of specimens, including blood and tissue donations, derived from the human body, solely or principally for the purpose of providing information on one or more of the following:<br>(a) concerning a physiological or pathological process or state;<br>(b) concerning congenital physical or mental impairments;<br>(c) concerning the predisposition to a medical condition or a disease;<br>(d) to determine the safety and compatibility with potential recipients;<br>(e) to predict treatment response or reactions;<br>(f) to define or monitoring therapeutic measures.<br>specimen receptacles shall also be deemed to be in vitro diagnostic medical devices. [3]

Table S2. Endings conditions after each iteration of the taxonomy development process.

**Ending conditions**	**Iteration 1: Conceptual-to-empirical**	**Iteration 2: Empirical-to-conceptual**	**Iteration 3: Evaluation**	
O = Objective ending condition, S = Subjective ending condition	Systematic literature review	Analysis of real-world business models	Experts interviews	Intercoder reliability
O1: All objects or a representative sample of objects have been examined	x	x	x	x
O2: No object was merged with similar objects or split into multiple objects in the last iteration				x
O3: At least one object is classified under every characteristic of every dimension		x	x	x
O4: No new dimensions or characteristics were added in the last iteration				x
O5: No dimensions or characteristics were merged or split in the last iteration				x
O6: Every dimension is unique and not repeated (i.e., there is no dimension duplication)	x	x	x	x
O7: Every characteristic is unique within its dimension (i.e., there is no characteristic duplication within a dimension)	x	x	x	x
O8: Each cell (Combination of characteristics) is unique and is not repeated (i.e., there is no cell duplication)	x	x	x	x
S1: Concise		x	x	x
S2: Robust		x	x	x
S3: Comprehensive		x	x	x
S4: Extendible	x	x	x	x
S5: Explanatory		x	x	x

Table S3. Resulting literature corpus after systematic literature review.

Author	Year	Objective	Methods	Core dimensions mentioned related to digital health and business models
Peters et al. [4]	2015	Design a telemedicine business model framework	Mixed-methods	Overall purpose, end consumer, partner network, realization of benefits for patients, portfolio role, contact with patient, domain-specific know-how, Required responsiveness, required means of communication, type of revenue, paying entity, cost drivers
Gehde et al. [5]	2022	Identify key configurations of digital health business models in Germany	Quantitative	Area of activity, digital technology
Oderanti et al. [6]	2021	Identify barriers and challenges and develop a business model framework	Qualitative	Key activities and value proposition, channels, key resources, key partners, customer segments, cost structure, customer relationship management, revenue streams, products and services
Acheampong and Vimerlund [7]	2015	Design a business model framework for telemedicine services	Conceptual	Outcomes, user group, facility, actors and roles, resources, revenue
Pascarelli et al. [8]	2023	Highlight current characteristics of digital health business models	Conceptual	Impact, revenue model, costs, funding, principal stakeholders, organizational activities, technology, legal and regulatory framework, key resources
Oderanti and Li [9]	2018	Identify barriers and challenges in eHealth and develop a business model framework	Qualitative	Product/service innovation, value propositions, financial viability, customer relationship management, stakeholder credibility, infrastructure management
Kijl and Nieuwenhuis [10]	2011	Design a business model for deploying an e-health service innovation	Mixed-methods	Value network, roles, revenue streams, regulations, technology, costs
Kijl et al. [11]	2010	Design a business model for deploying an e-health service innovation	Mixed-methods	Value network, roles, revenue streams, regulations, technology, costs
Garrot and Angele-Halgrand [12]	2022	Proposing a new business model typology for digital health	Conceptual	Offer/value proposition, resources, finance/profit formula, client/process
Böhler et al. [13]	2022	Analyze the transformational potential of digital health business models	Conceptual	Offering, patient journey, value chain, financial model
Tariq [14]	2023	Conceptualizing forces that impact business model innovation in digital health	Conceptual	Players, funding, policy, technology, customers, accountability
Mueller [15]	2019	Develop a mHealth business model framework for the maternal and baby segment	Mixed-methods	Target customer, market segment, servie offering, value proposition, mHealth category, regulation, mHealth intervention point, digital technology, key activities, key revenue stream, revenue source, price mechanism
Sterling and LeRouge [16]	2019	Identify core components of emerging business models for on-demand telemedicine	Qualitative	Value proposition, key processes, key resources, profit formula
Korsgaard et al. [17]	2021	Identify qualitative characteristics of business models of long-term operational telemedicine providers	Qualitative	Customer segments, value propositions, channels, customer relations, key resources, key activities, key partners

Continued from previous page				
**Author**	**Year**	**Objective**	**Methods**	**Core dimensions mentioned related to digital health and business models**
Velayati et al. [18]	2022	Develop a telehealth business framework for Iran	Mixed-methods	Making contracts, product marketing, product protection, responsibility of telehealth services, product evaluation, business initial requirements, value proposition, revenue model, revenue making, pricing, cost structure, customers and users, support services, market, stakeholders, licenses and permissions, key partners, key activities, key resources
Lin et al. [19]	2010	Analyze the business model of a telecardiology service	Mixed-methods	Value proposition, customer relationship, target customer, channels, value configuration, capability, partnership, cost structure, revenue model
Chen et al. [20]	2013	Study business models of eight telemedicine ventures	Qualitative	Customer segments, value propositions, channels, customer relations, key resources, key activities, key partnerships, cost structure
Alnadhi [21]	2023	Scrutinize different features of digital health business models	Conceptual	Value propositions, customer segments, customer relationships, revenue streams, key partners, key activities, key resources, channels, cost structure
Christie et al. [22]	2021	Examine a sample of case studies of eHealth interventions and derive lessons learned	Mixed-methods	Health system integration, key resources, key partners, cost structure, revenue structures, pricing
Tulu et al. [23]	2005	Classify telemedicine efforts and identify patterns	Quantitative	Application area, application purpose, delivery option, communication infrastructure, environmental setting
Harst et al. [24]	2022	Develop a telemedicine taxonomy	Quantitative	Application type, personnel involved, target population, setting, technology, data provision, intended outcome
Greve et al. [25]	2020	Develop a taxonomy and archetypes of mobile health applications	Mixed-methods	Location, transaction, registration, notification, data connectivity, communication, community, user, knowledge transfer, health management, health organization, data capture, appointment management, therapy
Glöggler and Ammenwerth [26]	2021	Validate a taxonomy of patient portals	Quantitative	Portal design, management, communication, instructions, self-management, self-determination, data management
Olla and Shimskey [27]	2015	Propose a taxonomy of existing and emerging mHealth applications	Qualitative	Medical use cases, technical modalities, policy considerations
Lehmann et al. [28]	2021	Present the mHealth atlas platform	Mixed-methods	Purpose of use, application area, expertise, medical application, term of application, operation mode, accessibility
Bashshur et al. [29]	2011	Present a taxonomy for telemedicine	Conceptual	Functionality, applications, technology
Ropposch and Rachinger [30]	2023	Develop business model types based on a taxonomy for mHealth in maternal and baby care	Mixed-methods	mHealth intervention point, key mHealth purpose, domain-specific know-how, cocreation role, portfolio role, service offering, target customer, key value proposition, mHealth category, key revenue stream, revenue source, price mechanism

Table S4. Selected digital health technology companies, including names, websites, and countries.

ID	Company	Website	Country
C1	Aaron	aaron.ai	Germany
C2	Aasa Health	vitala.health	Sweden
C3	AGGFox	pillio.de	Germany
C4	Aicura medical	aicura-medical.com	Germany
C5	Aignostics	aignostics.com	Germany
C6	Aiosyn	aiosyn.com	Netherland
C7	Alex Therapeutics	alextherapeutics.com	Sweden
C8	AlgoDx	algodx.com	Sweden
C9	Allisone Technology	allisone.ai	France
C10	Amie Technologies	palhelps.com	Netherlands
C11	Apotekamo	apotekamo.rs	Sweden
C12	ArtiQ B	artiq.eu	Belgium
C13	Aumio	aumio.de	Germany
C14	Avelios Medical	avelios.com	Germany
C15	Avi Medical Operations	avimedical.com	Germany
C16	Awell Health	awellhealth.com	Belgium
C17	BrainScan	www.brainscan.ai	Poland
C18	CardioSignal	www.cardiosignal.com	Finland
C19	Cardisio	cardis.io	Germany
C20	Care to translate	caretotranslate.com	Sweden
C21	Cerebriu	www.cerebriu.com	Denmark
C22	Charles	charles.co	France
C23	Chino	chino.io	Italy
C24	Clare&me	clareandme.com	Germany
C25	Climedo Health	climedo.de	Germany
C26	Zymego	zymego.com	Sweden
C27	Collective Minds Radiology	cmrad.com	Sweden
C28	Concilio	concilio.com	France
C29	CURE Group	getcure.app	Germany
C30	Cureety TechCare	cureety.com	France
C31	Deepc	deepc.ai	Germany
C32	Dermanostic	dermanostic.com	Germany
C33	Digital Mind Solutions	ifeelonline.com	Spain
C34	Doctor One	doctor.one	Poland
C35	Doctorly	doctorly.de	Germany
C36	Doctrio	doctrio.com	France
C37	Docyet	docyet.com	Germany
C38	Ecovery	ecovery.de	Germany
C39	Endo Health	endometriose.app	Germany
C40	enGenome	engenome.com	Italy
C41	eTherapists	humanoo.com	Germany
C42	Exakt Health	exakthealth.com	Germany
C43	Famedly	famedly.com	Germany
C44	Fastic	fastic.com	Germany

Continued from previous page			
**ID**	**Company**	**Website**	**Country**
C45	Fimo Health	fimohealth.com	Germany
C46	Floy	floy.com	Germany
C47	Formel Skin	formelskin.de	Germany
C48	Fosanis	mikahealth.com	Germany
C49	GET.ON	hellobetter.de	Germany
C50	Goreha	caspar-health.com	Germany
C51	Healee	healee.com	Bulgaria
C52	Heartbeat Medical	heartbeat-med.com	Germany
C53	Hedia	hedia.co	Denmark
C54	Hi.health	hi.health	Austria
C55	HiDoc Technologies	cara.care	Germany
C56	Hublo	hublo.com	France
C57	Idoven	idoven.ai	Spain
C58	ImVitro	im-vitro.com	France
C59	Incepto Medical	incepto-medical.com	France
C60	inHEART	inheartmedical.com	France
C61	IOMED	iomed.es	Spain
C62	Jutro Medical	jutromedical.com	Poland
C63	Kranus Health	kranushealth.com	Germany
C64	Labplus	labplus.pl	Poland
C65	Levvel Health	levvel.health	Denmark
C66	Lifen	lifen.fr	France
C67	Likeminded	likeminded.care	Germany
C68	Lindera	lindera.de	Germany
C69	Liva Healthcare	livahealthcare.com	Denmark
C70	LN Care	may-sante.com	France
C71	Lullaai Networks	lullaai.com	Spain
C72	LynxCare Clinical Informatics	lynx.care	Belgium
C73	MAYD Group	getmayd.com	Germany
C74	Medicus	medicus.ai	Austria
C75	MediQuo	mediquo.com	Spain
C76	Meditopia	meditopia.com	Germany
C77	MedoSync	medosync.com	Ireland
C78	Medsavana	savanamed.com	Spain
C79	Medwing	medwing.com	Germany
C80	Milvue	milvue.com	France
C81	MindAhead	mindahead.info	Germany
C82	Mindgram	mindgram.com	Poland
C83	Mindpeak	mindpeak.ai	Germany
C84	Moka.care	moka.care	France
C85	MX Labs	mxlabs.ai	Estonia
C86	Myosotis	myo.de	Germany
C87	Nabla Technologies	nabla.com	France
C88	NeoPrediX	neopredix.com/de	Germany
C89	Neotiv	neotiv.com	Germany

Continued from previous page			
**ID**	**Company**	**Website**	**Country**
C90	Neuroventis	neuroventis.care	Belgium
C91	Nilo.health	nilohealth.com	Germany
C92	Nostos Genomics	nostos-genomics.com	Germany
C93	Nova Leah	novaleah.com	Ireland
C94	Nyra Health	nyra.health	Austria
C95	OASYS NOW	oasysnow.com	Netherlands
C96	Oneleaf	oneleafhealth.com	France
C97	ONVY HealtTech Group	onvy.health	Germany
C98	OpenUp	openup.nl	Netherlands
C99	Orbit Health	orbit.health	Germany
C100	Oxipit	oxipit.ai	Lithuania
C101	patientMpower	patientmpower.com	Ireland
C102	PEEK HEALTH	peekmed.com	Portugal
C103	Poppins	poppins.io	France
C104	Powerful Medical	powerfulmedical.com	Slovakia
C105	Predict4Health	predict4health.com	France
C106	Previa Medical	previa-medical.com	France
C107	Prosoma	prosoma.com	Germany
C108	Qompium	fibricheck.com	Belgium
C109	Quenomedical	qunomedical.com	Germany
C110	RAYLYTIC	raylytic.com	Germany
C111	Recare	recaresolutions.com	Germany
C112	Resilience	resilience.care	France
C113	ScientiCore	hypherdata.com	Netherlands
C114	ScreenPoint Medical	screenpoint-medical.com	Netherlands
C115	Sêmeia	semeia.io	France
C116	Smart Reporting	smart-reporting.com	Germany
C117	Solve.Care	solve.care	Estonia
C118	Sonio	sonio.ai	France
C119	Steto	steto.care	France
C120	Stratipath	stratipath.com	Sweden
C121	Sunrise	hellosunrise.com	Belgium
C122	Sympatient	sympatient.com	Germany
C123	Synapse Medicine	synapse-medicine.com	France
C124	Telemedi	business.telemedi.com/en	Poland
C125	Tonic App	tonicapp.io	Portugal
C126	Tucuvi Care	tucuvi.com	Spain
C127	UpHill	uphillhealth.com	Portugal
C128	Vantis	vantis-health.com	Germany
C129	Vara	vara.ai	Germany
C130	Virtonomy	virtonomy.io	Germany
C131	VisionHealth	visionhealth.gmbh	Germany
C132	Vitadio	vitad.io	Czech Republic
C133	VitaDX	vitadx.com/en	France
C134	Wellbeing OS Ventures	moonoa.fr	Spain

Continued from previous page			
**ID**	**Company**	**Website**	**Country**
C135	Wellster Healthtech Group	wellsterhealth.com	Germany
C136	Whispp	whispp.com	Netherlands
C137	WhiteLab Genomics	whitelabgx.com	France
C138	XO Life	xo-life.com	Germany
C139	Xund	xund.ai	Hungary
C140	xWave Technologies	xwave.ie	Ireland
C141	Yuca	yuka.io	France
C142	Zerintia HealthTech	zerintiahealthtech.com	Spain

Table S5. Taxonomy revisions after the evaluation of the taxonomy through expert interviews.

**Taxonomy version before evaluation**	**Operation***	**Taxonomy version after evaluation**
Digital main purpose	Rename	Main purpose
Administrative support	-	Administrative support
Big data analytics	Delete	-
Clinical research support	-	Clinical research support
Communication or mentoring	Rename	Telecommunication
Diagnosis or prediction	-	Diagnosis or prediction
Health or lifestyle e-commerce	Merging	Online marketplace or matchmaking support
Information delivery or education	-	Information delivery or education
Monitoring support	-	Monitoring support
Search or matching support	Merging	Online marketplace or matchmaking support
Solution acceleration	Rename	Provide raw technology
Therapy or interventional purpose	Rename	Treatment or interventional purpose
Target user	-	Target user
Health insurer	Rename	Health insurer or government
Health care provider	-	Health care provider
Patient or private individual	-	Patient or private individual
Pharmaceutical company	Merging	Indirect health care commercial firm
Research institution	-	Research institution
Technology company	Merging	Indirect health care commercial firm
-	Add	Non-health care commercial firm
Clinical evidence	Rename	Evidence
Irrelevant	Delete	-
Evidence backed	Rename	Evidence based
Undemonstrated	Rename	Nonevidence based
User offering	Rename	User interface
Application programming interface	Rename	Developer tool
Desktop app	Rename	Desktop application
Mobile or web app	Rename	Mobile app or web application
Software extension	-	Software extension
Medical specialty level		Level of care
Level-neutral	Rename	Outside medical care
Primary care	-	Primary care
Secondary care	-	Secondary care
Tertiary care	-	Tertiary care
Stage of care cycle	Delete	-
Aftercare	Delete	-
Incare	Delete	-
Precare	Delete	-
Stage-neutral	Delete	-

Continued from previous page

**Taxonomy version before evaluation**	**Operation***	**Taxonomy version after evaluation**
Medical device regulatory framework	-	Medical device regulatory framework
In vitro diagnostic medical device regulation	-	In vitro diagnostic medical device regulation
Medical device regulation	-	Medical device regulation
Nonmedical device	-	Nonmedical device
Data type	-	Data type
Biological data	Rename	Human biological material data
Laboratory or medical test data	Rename	Medical laboratory results data
Nondata driven	Rename	Nonspecific data
Multimedia data	Rename	Educational multimedia data
Organizational data	-	Organizational data
Optical or medical imaging data	Rename	Medical imaging data
Patient-reported outcome data	Rename	User activity or patient-reported data
Pharmacological data	Rename	Medication data
Vital parameter data	Rename	Physiological parameter data
Enabling technology	-	Enabling technology
Artificial intelligence	-	Artificial intelligence
Cloud technology	Rename	Cloud or network technology
Data security or protection technology	-	Data security or protection technology
Nonspecific technology	-	Nonspecific technology
Sensor technology	-	Sensor technology
Virtual or augmented reality	-	Virtual or augmented reality
Paying entity	-	Paying entity
Corporate employer	Rename	Non-health care commercial firm
Health insurer	Rename	Health insurer or government
Health care provider	-	Health care provider
Patient or private individual	-	Patient or private individual
Pharmaceutical company	Merging	Indirect health care commercial firm
Research institution	-	Research institution
Technology company	Merging	Indirect health care commercial firm
Reimbursement	-	Reimbursement
Nonreimbursable	-	Nonreimbursable
Reimbursable	-	Reimbursable
Platform interaction type	Rename	Stakeholder interaction type
Interuser interaction	-	Interuser interaction
In-house user interaction	Rename	Solution provider-user interaction
Nonplatformized	Rename	Nonuser Interaction

* Operations: *add* (insert a new element), *rename* (change the name of an element), *merging* (combine existing elements), and *delete* (remove an existing element)

Figure S1. The dendrogram, based on hierarchical clustering using Ward's method, built from data on 169 real-world digital health technology business models.

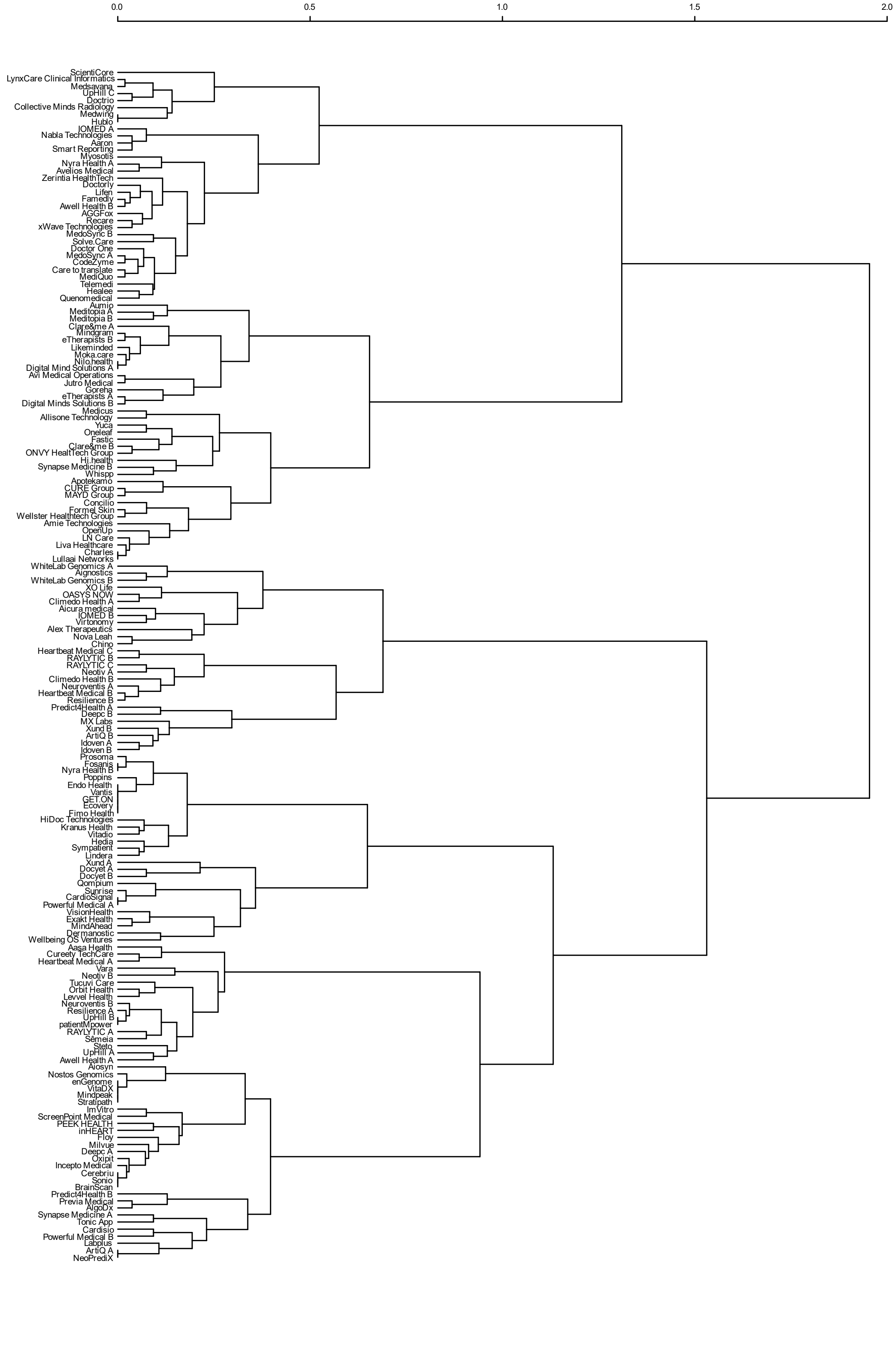

Figure S2. Elbow plot for k-means clustering.

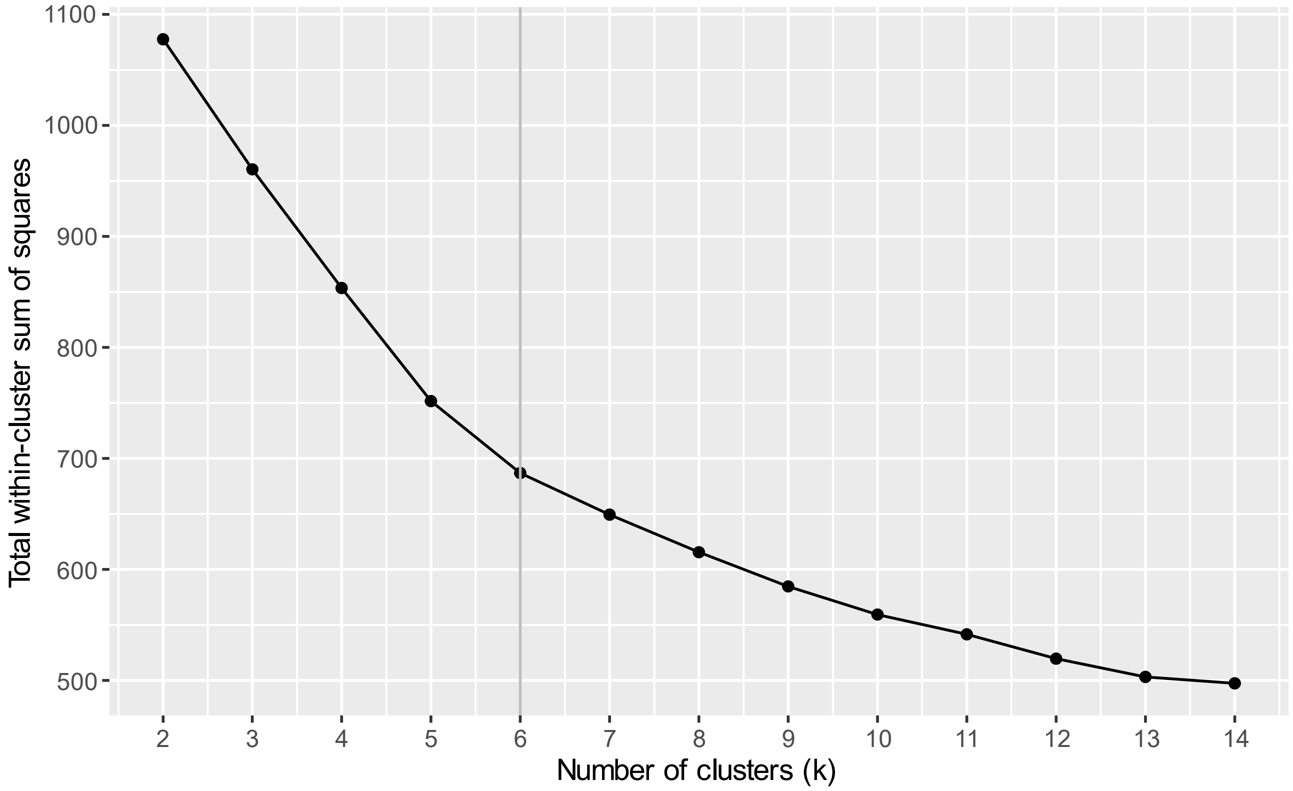

Table S6. Cluster metrics comparisons for different number of cluster solutions.

**Number of cluster solutions**	**3**	**4**	**5**	**6**	**7**	**8**	**9**	**10**
Theoretical equal cluster size[a]	56	42	34	28	24	21	19	17
Actual cluster sizes[b]	64, 50, 55	37, 34, 69, 29	49, 29, 33, 37, 21	35, 21, 34, 18, 28, 33	35, 18, 33, 21, 17, 12, 33	14, 14, 21, 14, 34, 23, 18, 31	18, 17, 14, 22, 11, 19, 25, 31, 12	20, 25, 14, 11, 12, 18, 25, 11, 13, 20
Number of clusters supported by dendrogram[c]	yes	yes	yes	yes	no	no	no	no
Number of clusters supported by elbow rule[d]	no	no	no	yes	no	no	no	no

[a] Theoretical number of cases per cluster if all clusters have equal size (total number of cases divided by number of clusters)
[b] Number of cases per cluster after k-means clustering, where small sample sizes might be difficult to interpret in archetype analysis
[c] Clusters merging at higher dendrogram heights are more dissimilar, suggesting distinct archetypes with significant differences
[d] Elbow point where reduction of within-cluster sum of squares slows down indicates the optimal number of clusters

Table S7. Frequency distribution of characteristics for the six digital health technology business model archetypes.

Dimension	Characteristic	A1	A2	A3	A4	A5	A6
Number of cases per cluster		35	21	34	18	28	33
Main purpose	Administrative support	94%	0%	15%	28%	7%	9%
	Clinical research support	0%	0%	3%	0%	75%	0%
	Telecommunication	34%	14%	6%	50%	0%	88%
	Diagnosis or prediction	0%	0%	88%	28%	36%	3%
	Information delivery or education	14%	5%	6%	44%	7%	88%
	Monitoring support	3%	5%	9%	89%	32%	0%
	Online marketplace or matchmaking support	17%	0%	6%	0%	14%	12%
	Provide raw technology	0%	0%	0%	0%	29%	0%
	Treatment or interventional purpose	0%	95%	12%	6%	11%	0%
Target user	Health insurer or government	6%	0%	6%	0%	0%	6%
	Health care provider	97%	5%	79%	100%	0%	9%
	Indirect health care commercial firm	0%	0%	3%	0%	61%	0%
	Non-health care commercial firm	0%	0%	0%	0%	4%	21%
	Patient or private individual	46%	100%	26%	94%	25%	100%
	Research institution	0%	0%	0%	0%	36%	0%
Evidence	Evidence based	0%	100%	100%	100%	50%	12%
	Nonevidence based	100%	0%	0%	0%	50%	88%
Level of care	Outside medical care	20%	0%	0%	0%	100%	0%
	Primary care	63%	86%	41%	28%	0%	94%
	Secondary care	66%	81%	76%	83%	0%	9%
	Tertiary care	63%	19%	68%	89%	0%	9%
Medical device regulatory framework	In vitro diagnostics medical device regulation	0%	0%	18%	0%	0%	0%
	Medical device regulation	0%	95%	82%	89%	54%	3%
	Nonmedical device	100%	5%	0%	11%	46%	97%
Data type	Educational multimedia data	3%	95%	0%	6%	11%	70%
	Human biological material data	0%	0%	21%	0%	11%	0%
	Medical laboratory results data	0%	0%	18%	6%	11%	3%
	Nonspecific data	0%	0%	0%	0%	7%	0%
	Medical imaging data	6%	0%	32%	11%	18%	0%
	Organizational data	89%	0%	6%	22%	7%	12%
	Physiological parameter data	0%	0%	24%	17%	21%	3%
	Medication data	9%	0%	6%	0%	0%	6%
	User activity or patient-reported data	17%	76%	6%	94%	57%	45%
Enabling technology	Artificial intelligence	34%	19%	91%	28%	61%	21%
	Cloud or network technology	83%	19%	12%	94%	46%	88%
	Data security or protection technology	9%	0%	0%	0%	7%	0%
	Nonspecific technology	0%	57%	0%	0%	4%	9%
	Sensor technology	14%	10%	18%	17%	4%	52%
	Virtual reality or augmented reality	0%	5%	3%	0%	0%	0%

Continued from previous page								
**Dimension**	**Characteristic**	**A1**	**A2**	**A3**	**A4**	**A5**	**A6**
Paying entity	Health insurer or government	3%	81%	9%	11%	0%	18%
	Health care provider	89%	0%	76%	94%	0%	3%
	Indirect health care commercial firm	0%	0%	3%	0%	61%	0%
	Non-healthcare commercial firm	0%	0%	0%	0%	4%	24%
	Patient or private Individual	11%	24%	15%	6%	0%	58%
	Research Institution	0%	0%	0%	0%	36%	0%
Reimbursement	Nonreimbursable	100%	19%	97%	78%	100%	58%
	Reimbursable	0%	81%	3%	22%	0%	42%
User interface	Developer tool	9%	0%	9%	0%	25%	0%
	Desktop application	6%	0%	6%	0%	0%	0%
	Mobile app or web application	97%	100%	91%	100%	82%	100%
	Software extension	6%	0%	15%	0%	4%	9%
Stakeholder interaction type	Interuser interaction	71%	5%	6%	100%	43%	6%
	Nonuser interaction	29%	81%	91%	0%	57%	30%
	Solution provider-user interaction	6%	14%	3%	0%	0%	64%

Legend

0 – 30 %	31 - 50%	51 - 80%	81 - 100%

Figure S3. The business model taxonomy for digital health technology ventures in the European Union. *E=exclusive dimension (only 1 characteristic assignable) **N=nonexclusive dimension (multiple characteristics assignable)

	Dimension	Characteristics									E*/N**
Value proposition	Main purpose	Administrative support	Clinical research support	Telecommunication	Diagnosis or prediction	Information delivery or education	Monitoring support	Online marketplace or matchmaking support	Provide raw technology	Treatment or interventional purpose	N
	Target user	Health insurer or government	Health care provider	Indirect health care commercial firm	Non–health care commercial firm	Patient or private individual	Research institution				N
	Evidence	Evidence based	Nonevidence based								E
Value architecture	Level of care	Outside medical care	Primary care	Secondary care	Tertiary care						N
	Medical device regulatory framework	In vitro diagnostic medical device regulation	Medical device regulation	Nonmedical device							E
	Data type	Educational multimedia data	Human biological material data	Medical laboratory results data	Nonspecific data	Medical imaging data	Organizational data	Physiological parameter data	Medication data	User activity or patient-reported data	N
	Enabling technology	Artificial intelligence	Cloud or network technology	Data security or protection technology	Nonspecific technology	Sensor technology	Virtual or augmented reality				N
Value finance	Paying entity	Health insurer or government	Health care provider	Indirect health care commercial firm	Non–health care commercial firm	Patient or private individual	Research institution				N
	Reimbursement	Nonreimbursable	Reimbursable								E
Value network	User interface	Desktop application	Developer tool	Mobile app or web application	Software extension						N
	Stakeholder interaction type	Interuser interaction	Nonuser interaction	Solution provider–user interaction							N

Table S8. Detailed description and definition of each dimension and characteristic of the digital health business model taxonomy.

**Main purpose**	**What is the main purpose that drives the value proposition?**
Administrative support	Support in organizational matters (e.g., management of appointments, financials, data organization)
Clinical research support	Support in digital management and execution of clinical trials
Telecommunication	Provide general communication channels or use communication channels for coaching
Diagnosis or prediction	Directly diagnosing or supporting with the diagnosis and prediction of medical conditions (medical purpose)
Information delivery or education	Delivery of information or education without a specific medical purpose
Monitoring support	Collect and present data that helps to monitor medical conditions (medical purposes)
Online marketplace or matchmaking support	Enable digital offering and selection of solutions or create matches between interested parties
Provide raw technology	Provide technology that enhances third-party companies' technological solution
Treatment or interventional purpose	Support in treatment, prevention, alleviation, or compensation of medical conditions through an intervention or in planning or execution of such an intervention (medical purpose) (e.g., digital cognitive behavioral therapy, rehabilitation)
**Target user**	**Who is the target user that will mainly use the solution?**
Health insurer or government	Private or public health insurers that provide financial coverage for medical expenses
Health care provider	Any entity that provides direct health care (e.g., physicians, hospitals, nurses, pharmacies)
Indirect health care commercial firm	Any kind of private businesses that have an indirect impact on healthcare, such as technology companies (e.g., medical technology companies) or pharmaceutical companies
Non-health care commercial firm	Any private business that is not directly or indirectly involved in healthcare
Patient or private individual	Patients or private individuals who might not (yet) be patients (e.g., employees, elderly)
Research institution	Organizations dedicated to conducting academic research
**Evidence**	**Was data collected that proves that the solution works as promised (clinical and non-clinical evidence)?**
Evidence based	Data was collected that proves safety, performance, or clinical benefits (e.g., published peer-reviewed studies)
Nonevidence based	No evidence data was collected
**Level of care**	**In which stage of the health care system is the underlying solution integrated?**
Outside medical care	Solution applied outside of health care (e.g., research application)
Primary care	Usage during first contact point with health care, often health care providers act as the first contact point and principal point for coordination of care (e.g., family physician, pharmacist, psychotherapist)
Secondary care	Usage in acute, short-term, and sporadic consultation from a specialist to provide expert opinion and/or surgical or other advanced interventions (e.g., specialist treatment, short-term hospitalization, routine surgery, medical imaging, rehabilitation)
Tertiary care	Usage in health care that is institution-based, highly specialized, and technology-driven or focused on long-term care (e.g., open heart surgery, burn treatment, elderly care)

Continued from previous page	
**Medical device regulatory framework**	**Under which EU medical device regulatory framework does the solution of the business model fall?**
In vitro diagnostic medical device regulation	Solution falls under In Vitro Diagnostic Medical Device Regulation (i.e.., in vitro diagnostics with a medical purpose using data from human biological samples)
Medical device regulation	Solution falls under Medical Device Regulation (e.g., solutions with a medical purpose except in vitro diagnostics)
Nonmedical device	Solution does not fall under EU medical device regulations
**Data type**	**Which type of input or output data is driving the business model?**
Educational multimedia data	Multimedia data used for education of users (e.g., text, video, audio)
Human biological sample data	Data from biological samples derived from the human body (e.g., microscopic cell data, genomic data)
Medical laboratory results data	Data from laboratory results or medical tests (e.g., blood values, breathing test results)
Nonspecific data	No specific data type is identifiable
Medical imaging data	Data in the form of medical images (e.g., X-ray scans, MRI images, ultrasound images)
Organizational data	Data related to the organization of health care (e.g., appointment, billing, communication)
Physiological parameter data	Data obtained from measurement of bodily functions (e.g., pulse, blood pressure, respiratory rate)
Medication data	Data related to drugs and medications (e.g., drug information, drug interaction)
User activity or patient-reported data	Data related to tracking of user activities, user sensory data, personal health profile (e.g., physical activity, food tracking, demographics), and patient-reported outcomes often related to treatment success
**Enabling technology**	**Through which digital technology is the business model and the value proposition primarily enabled?**
Artificial intelligence	Enabled through different types of artificial intelligence (e.g., deep learning)
Cloud or network technology	Enabled through remote computation and data transfer
Data security or protection technology	Digital technology related to data security and protection (e.g., blockchain)
Nonspecific technology	Business model is primarily enabled through the user interface
Sensor technology	Enabled through different sensor types (e.g., microphone, camera, or pulse sensor)
Virtual reality or augmented reality	Digital technology related to digital environments or overlaying digital information onto the real world
**Paying entity**	**Who is paying for the solution?**
Health insurer or government	Health insurers that provide financial coverage for medical expenses or government
Health care provider	Any entity that provides direct health care (e.g., physicians, hospitals, nurses, pharmacies)
Indirect health care commercial firm	Any kind of private businesses that have an indirect impact on health care, such as technology companies (e.g. medical technology companies) or pharmaceutical companies
Non-health care commercial firm	Any private business that is not directly or indirectly involved in health care
Patient or private individual	Patients or private individuals who might not (yet) be patients (e.g., employees, elderly)
Research institution	Organizations dedicated to conducting academic research
**Reimbursement**	**Are the costs for solution usage compensated?**
Reimbursable	Costs are compensated by a third party, mostly through health insurer but also corporate employers
Nonreimbursable	Costs are not compensated by a third party

Continued from previous page

User interface	Through which interface is the solution offered to the user?
Developer tool	Tools such as application programming interfaces or software development kits
Desktop application	A software solution that is installed on a desktop computer and runs on the operating system
Mobile app or web application	A software application that is accessible through internet browsers or can be installed on mobile devices
Software extension	A piece of software designed to augment the capabilities of a primary software application
**Stakeholder interaction type**	**Which type of stakeholder interaction is the business model based on?**
Interuser interaction	Users interact with each other through the platform (e.g., patient interacts with health care provider, patient interacts with patient, users exchange services)
Solution provider-user interaction	Solution provider interact with users through the platform (e.g., users can consult coaches employed by solution provider, internal digital marketplace of solution provider offerings)
Nonuser interaction	Solution does not connect users and therefore is not a platform

Table S9. Exemplary application of the business model taxonomy for digital health technology ventures within the European Union.

Exemplary usage of the business model taxonomy with black fields as selected characteristics for the use case

	Dimension	Characteristics						E*/N**
Value proposition	Main purpose	Administrative support	Clinical research support	Telecommunication	**Diagnosis or prediction**	Information delivery or education		N
		Monitoring support	Online marketplace or matchmaking support	Provide raw technology	Treatment or interventional purpose			
	Target user	Health insurer or government	**Health care provider**	Indirect health care commercial firm	Non–health care commercial firm	Patient or private individual	Research institution	N
	Evidence	**Evidence based**	Nonevidence based					E
Value architecture	Level of care	Outside medical care	Primary care	**Secondary care**	**Tertiary care**			N
	Medical device regulatory framework	In vitro diagnostic medical device regulation	**Medical device regulation**	Nonmedical device				E
	Data type	Educational multimedia data	Human biological material data	Medical laboratory results data	Nonspecific data	**Medical imaging data**		N
		Organizational data	Physiological parameter data	Medication data	User activity or patient-reported data			
	Enabling technology	**Artificial intelligence**	Cloud or network technology	Data security or protection technology	Nonspecific technology	Sensor technology	Virtual or augmented reality	N
Value finance	Paying entity	Health insurer or government	**Health care provider**	Indirect health care commercial firm	Non–health care commercial firm	Patient or private individual	Research institution	N
	Reimbursement	**Nonreimbursable**	Reimbursable					E
Value network	User interface	Desktop application	Developer tool	**Mobile app or web application**	Software extension			N
	Stakeholder interaction type	Interuser interaction	**Nonuser interaction**	Solution provider–user interaction				N

Description of the use case for a digital health technology business model

The example venture is a digital health technology company focused on developing machine learning solutions in medical imaging to support radiologists. Their software predicts whether a second MRI*** scan is needed and thereby supports radiologists in making timely decisions, minimizing the need for follow-up visits. This aligns with their value proposition, which has a medical purpose centered on diagnosis and prediction. Its primary target users are radiologists in the role of health care providers. Their business model is structured around a medical device due to its medical purpose, ensuring compliance with medical device regulation. Therefore, the company's value proposition is supported by collected clinical evidence. This business model is seamlessly integrated into health care systems within secondary and tertiary care settings. By leveraging artificial intelligence technology that relies on medical imaging data, the company generates revenue through direct payments from health care providers, making their solution non-reimbursable. The user interface is delivered through a web application, with no direct interaction between users taking place.

*E=exclusive dimension (only 1 characteristic assignable)

**N=nonexclusive dimension (multiple characteristics assignable)

***MRI=Magnetic Resonance Imaging

References

[1] International Organization for Standardization. ISO/TR 11147:2023 Health informatics - personalized digital health - digital therapeutics health software systems 2023.

[2] European Union. Regulation (EU) 2017/745 on medical devices. Official Journal of the European Union 2017;117:1-175.

[3] European Union. Regulation (EU) 2017/746 on in vitro diagnostic medical devices. Official Journal of the European Union 2017;117:176-332.

[4] Peters C, Blohm I, Leimeister JM. Anatomy of Successful Business Models for Complex Services: Insights from the Telemedicine Field. Journal of Management Information Systems 2015;32(3):75–104. [doi:10.1080/07421222.2015.1095034]

[5] Gehde KM, Rausch F, Leker J. BUSINESS MODEL CONFIGURATIONS IN DIGITAL HEALTHCARE—A GERMAN CASE STUDY ABOUT DIGITAL TRANSFORMATION. Int. J. Innov. Mgt. 2022;26(3):2240018. [doi:10.1142/S1363919622400187]

[6] Oderanti FO, Li F, Cubric M, Shi X. Business models for sustainable commercialisation of digital healthcare (eHealth) innovations for an increasingly ageing population. Technological Forecasting and Social Change 2021;171:120969. [doi:10.1016/j.techfore.2021.120969]

[7] Acheampong F, Vimarlund V. Business models for telemedicine services: a literature review. Health Systems 2015;4:189–203. [doi: 10.1057/hs.2014.20]

[8] Pascarelli C, Colucci C, Mitrano G, Corallo A. Business Models in Digital Health: Bibliometric Analysis and Systematic Literature Review. In: Proceedings of IEEE Symposium on Computers and Communications. 2023 Presented at: IEEE Symposium on Computers and Communications; July 9-12, Gammarth, Tunisia p. 1–4. [doi:10.1109/ISCC58397.2023.10218237]

[9] Oderanti FO, Li F. 2018. Commercialization of eHealth innovations in the market of the UK healthcare sector: A framework for a sustainable business model. Psychology and Marketing 2018;35:120–137. [doi: 10.1002/mar.21074]

[10] Kijl B, Nieuwenhuis LJM. Deploying e-health service innovations – an early stage business model engineering and regulatory validation approach. IJHTM 2011;12:23. [doi: 10.1504/IJHTM.2011.037219]

[11] Kijl B, Nieuwenhuis LJM, Huis In ’T Veld RMHA, Hermens HJ, Vollenbroek-Hutten MMR. Deployment of e-health services – a business model engineering strategy. J Telemed Telecare 2010;16:344–353. [doi: 10.1258/jtt.2010.006009]

[12] Garrot T, Angelé-Halgand N. Digital Health Business Models: Reconciling Individual Focus and Equity? In: Menvielle L, Audrain-Pontevia AF, Menvielle W, editors. The Digitization of Healthcare. 1st edition. London: Palgrave Macmillan UK; 2022. P. 59–78. [doi: 10.1057/978-1-349-95173-4_4]

[13] Böhler D. Digital Health Business Models: Transformation Is on the Horizon. In: Friebe M, editors. Novel Innovation Design for the Future of Health. 1st edition. Cham: Springer International Publishing; 2022. p. 153–160. [doi:10.1007/978-3-031-08191-0_15]

[14] Tariq MU. Healthcare Innovation & Entrepreneurship, Digital Health Entrepreneurship. In: Raimi L, Oreagba IA, editors, Medical Entrepreneurship. 1st edition. Singapore: Springer Nature Singapore; 2023. p. 243–258. [doi:10.1007/978-981-19-6696-5_16]

[15] Mueller C. mHealth Business Model Framework for the Maternal and Baby Segment: A Design Science Research Approach. In: Proceedings of 32nd Bled eConference. 2019 Presented at: 32nd Bled eConference Humanizing Technology for a Sustainable Society; June 16-19; Bled, Slovenia. [doi:10.18690/978-961-286-280-0.55]

[16] Sterling R, LeRouge C. On-Demand Telemedicine as a Disruptive Health Technology: Qualitative Study Exploring Emerging Business Models and Strategies Among Early Adopter Organizations in the United States. J Med Internet Res 2019;21:e14304. [doi:10.2196/14304]

[17] Korsgaard F, Hasenkam JM, Vesterby M. Successful implementation of telemedicine depends on personal relations between company representatives and healthcare providers: A qualitative study of business models for Danish home telemonitoring. Health Serv Manage Res 2021;34:223–233.

[18] Velayati F, Ayatollahi H, Hemmat M, Dehghan R. The 4P telehealth business framework for Iran. BMC Med Inform Decis Mak 2022;22:266. [doi:10.1186/s12911-022-02011-4]

[19] Lin SH, Liu JH, Wei J, Yin WH, Chen HH, Chiu WT. A Business Model Analysis of Telecardiology Service. Telemedicine and e-Health 2010;16:1067–1073. [doi:10.1089/tmj.2010.0059]

[20] Chen S, Cheng A, Mehta K. A Review of Telemedicine Business Models. Telemedicine and e-Health 2013;19(4):287–297. [doi:10.1089/tmj.2012.0172]

[21] Alnahdi S. An Psychological Mindset based Stress free Business Model for Digital Health. Journal for Re Attach Therapy and Developmental Diversities 2023;6(4s);401-412.

[22] Christie HL, Boots LMM, Hermans I, Govers M, Tange HJ, Verhey FRJ, De Vugt M. Business Models of eHealth Interventions to Support Informal Caregivers of People With Dementia in the Netherlands: Analysis of Case Studies. JMIR Aging 2021;4:e24724. [doi:10.2196/24724]

[23] Tulu B, Chatterjee S, Laxminarayan S. A Taxonomy of Telemedicine Efforts with Respect to Applications, Infrastructure, Delivery Tools, Type of Setting and Purpose. In: Proceedings of the 38th Annual Hawaii International Conference on System Sciences. 2005 Presented at: 38th Annual Hawaii International Conference on System Sciences; January 3-6; Big Island, USA. [doi:10.1109/HICSS.2005.56]

[24] Harst L, Otto L, Timpel P, Richter P, Lantzsch H, Wollschlaeger B, Winkler K, Schlieter H. An empirically sound telemedicine taxonomy – applying the CAFE methodology. J Public Health 2022;30:2729–2740. [doi:10.1007/s10389-021-01558-2]

[25] Greve M, Lembcke T-B, Diederich S, Brendel AB, Kolbe LM. Healthy by App – Towards a Taxonomy of Mobile Health Applications. In: Proceedings of 2020 PACIS. 2020 Presented at: PACIS; June 20-24; Dubai, United Arab Emirates.

[26] Glöggler M, Ammenwerth E. Improvement and Evaluation of the TOPCOP Taxonomy of Patient Portals: Taxonomy-Evaluation-Delphi (TED) Approach. J Med Internet Res 2021;23(10):e30701. [doi:10.2196/30701]

[27] Olla P, Shimskey C. mHealth taxonomy: a literature survey of mobile health applications. Health Technol. 2015;4:299–308. [doi:10.1007/s12553-014-0093-8]

[28] Lehmann, NJ, Spielmann F, George B, Ververs L, Karagulle MU, Kmiotek D, Mielke L, Junk O, Voisard A, Fluhr JW. mHealthAtlas - An Approach for the Multidisciplinary Evaluation of mHealth Applications. In: Proceedings of 2020 IEEE International Conference on E-Health Networking, Application & Services (HEALTHCOM). 2021 Presented at: 2020 IEEE International Conference on E-health Networking, Application & Services (HEALTHCOM); March 1-2; Shenzhen, China. [doi:10.1109/HEALTHCOM49281.2021.9399045]

[29] Bashshur R, Shannon G, Krupinski E, Grigsby J. The Taxonomy of Telemedicine. Telemedicine and e-Health 2011;17:484–494. [doi:10.1089/tmj.2011.0103]

[30] Ropposch C, Rachinger M, 2023. mHealth Business Model Types in Maternal and Baby Care - A Service-dominant Logic View. In: Academy of Management Proceedings. 2023 Presented at: 83rd Annual Meeting of the Academy of Management, August 4-8, Chicago, United States. [doi:10.5465/AMPROC.2023.17882abstract]
